# Simulations to Cover the Waterfront for Iron Oxide Catalysis

**DOI:** 10.1002/cphc.202200025

**Published:** 2022-02-15

**Authors:** Nadav Snir, Maytal Caspary Toroker

**Affiliations:** ^1^ Department of Materials Science and Engineering Technion - Israel Institute of Technology Haifa 3200003 Israel; ^2^ The Nancy and Stephen Grand Technion Energy Program Haifa Israel

**Keywords:** oxygen evolution reaction, water splitting, catalysis, Monte Carlo simulations, hematite

## Abstract

Hematite has been widely studied for catalytic water splitting, but the role of the interactions between catalytic sites is unknown. In this paper, we calculate the oxygen evolution reaction free energies and the surface adsorption distribution using a combination of density functional theory and Monte Carlo simulations to “cover the waterfront,” or cover a wide range of properties with a simulation of the hematite surface under working conditions. First, we show that modeling noninteracting catalytic sites provides a poor explanation of hematite's slow reaction kinetics. The interactions between the catalytic site may hinder catalysis through the strong interactions of *OH_2_ and *OOH intermediates, which cause the reaction to revert back to the *O intermediate. Hence, neighboring interactions may be a possible reason for the abundant, experimentally observed *O intermediate on the surface. This study demonstrates how neighboring sites impact the energy required for catalytic steps, thus providing new avenues to improve catalysis by controlling neighboring site interactions.

## Graphical abstract

**Interacting Neighbors**: A model of interacting surface terminations on neighboring sites shows new possible reaction pathways and a strong interaction of *OH_2_ and *OOH neighbors that provides a new mechanism responsible for the experimental observation identifying *O as the reaction bottleneck. This study uses a Monte Carlo simulation to show how neighbor interactions affect surface coverage of intermediates even when there is high applied voltage or pH level.

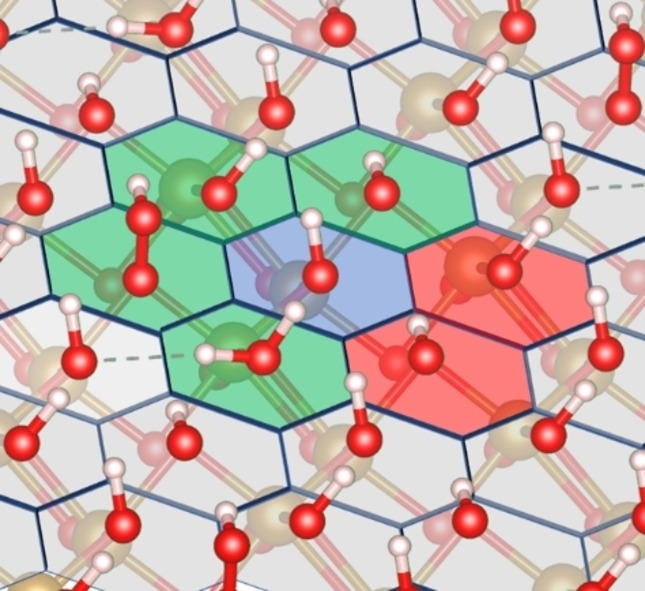

## Introduction

Hydrogen is an essential part of today's economy.[^1^, ^2^] Current hydrogen production methods are highly polluting,^[3]^ thus encouraging the search for cleaner production methods. One method for producing hydrogen is water splitting in an electrochemical cell (PEC).[^4^, ^5^, ^6^, ^7^] In a PEC, water is split into hydrogen and oxygen. The anode of the PEC produces oxygen by the oxygen evolution reaction (OER) or water oxidation.^[4]^ One of the biggest challenges in water splitting is finding an efficient catalyst for the OER. The oxygen evolution reaction requires the transfer of four electrons, making the ideal catalyst hard to find.^[4]^

Water oxidation in a PEC requires a catalyst. One of the most studied catalysts is α‐Fe_2_O_3_ or hematite.^[8]^ Hematite is stable under alkaline conditions and has a favorable band gap for water splitting but suffers from slow OER kinetics.^[8]^ Though there have been many studies of hematite, there is still more to understand, such as the interactions of termination species.

The OER under alkaline conditions can be modeled in five reactions:^[9]^

(1) $${}^{*}+H_{2}O\to {}^{{}^{*}}OH_{2}$$

(2) $${}^{{}^{*}}OH_{2}+OH^{-}+h^{+}\to H_{2}O+{}^{{}^{*}}OH$$

(3) $${}^{{}^{*}}OH+OH^{-}+h^{+}\to H_{2}O+{}^{{}^{*}}O$$

(4) $${}^{{}^{*}}O+OH^{-}+h^{+}\to {}^{{}^{*}}OOH$$

(5) $${}^{{}^{*}}OOH+OH^{-}+h^{+}\to O_{2}+H_{2}O+{}^{*}$$

where * denotes a surface vacancy and *X denotes an adsorbed surface species.

**Figure 1:**
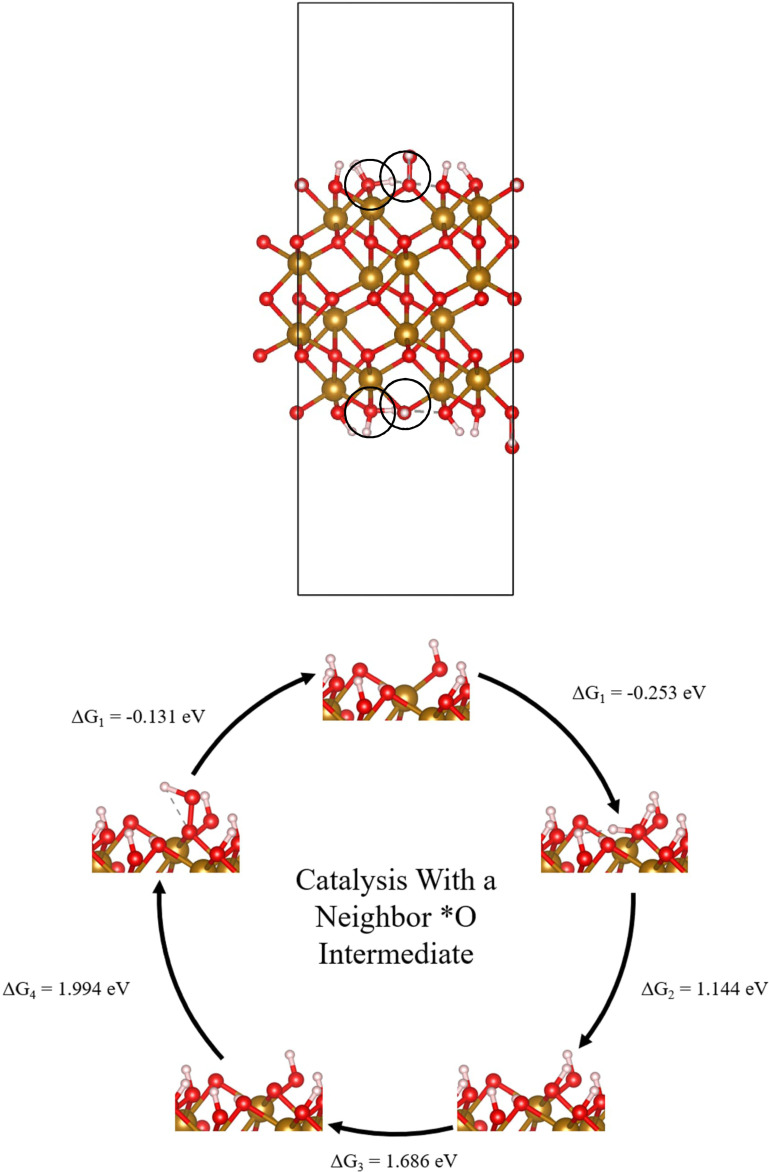
(top) Example slab of neighboring *OH_2_−*OOH sites. Surface *OH_2_ and *OOH sites circled. (bottom) An example cycle with *O as a neighbor and its free energy differences at pH=0.

Previous works concentrated on single‐site reactions[^10^, ^11^] and two‐site species interactions^[12]^ that form new intermediates and different reactions. First, previous works calculated the free energy differences between two consecutive reactive species. Later, larger slabs were used to show that when terminations are far apart, they do not significantly affect the calculated free energy.^[10]^ In this paper, we investigate the effect of near‐neighbor sites on catalysis using a computational density functional theory method and Monte Carlo simulations. Since switching the location of two species does not affect the total energy, the number of slabs was reduced from 25 to 15. The slabs were created using MATLAB by combining four smaller slabs comprised of the five reaction intermediates. In each 2×2 slab, two intermediates were connected to a single iron atom. All other sites were taken to be hydroxyl terminated *OH sites, since the hydroxyl termination is the initial intermediate before the onset of catalysis. An example slab is portrayed in Figure 1. The lattice constants of each slab were a=b=10.20 Å, c=24.28 Å, α=β=90°, and γ=120°. We used the (0001) surface of hematite, which is one of the natural growth surfaces of hematite.^[13]^

## Computational Details

Calculations were performed in VASP 5.4.4[^14^, ^15^, ^16^, ^17^] using the Perdew‐Burke‐Ernzerhof (PBE) exchange‐correlation functional. The spin‐polarized DFT+U approach by Dudarev et al.^[18]^ was used with a U−J value of 4.3 eV for iron, as was derived by ab initio methods.^[19]^ Values for oxygen and hydrogen were set to zero. Core electrons were described by projector augmented wave (PAW) potentials.

The Kohn‐Sham equations were solved self‐consistently using a plane‐wave basis and three‐dimensional periodic boundary conditions. K‐space integration was performed using the tetrahedron method with Blöchl correction.[^20^, ^21^] The plane‐wave energy cutoff was 700 eV. Gamma‐centered k‐grids of 2×2×1 were used for the slabs. These parameters converge the total energy to within 1 meV. Geometric relaxations were performed using the conjugate gradient (CG) method^[22]^ with a tolerance of 0.03 eV/Å for atomic forces.

**Figure 2:**
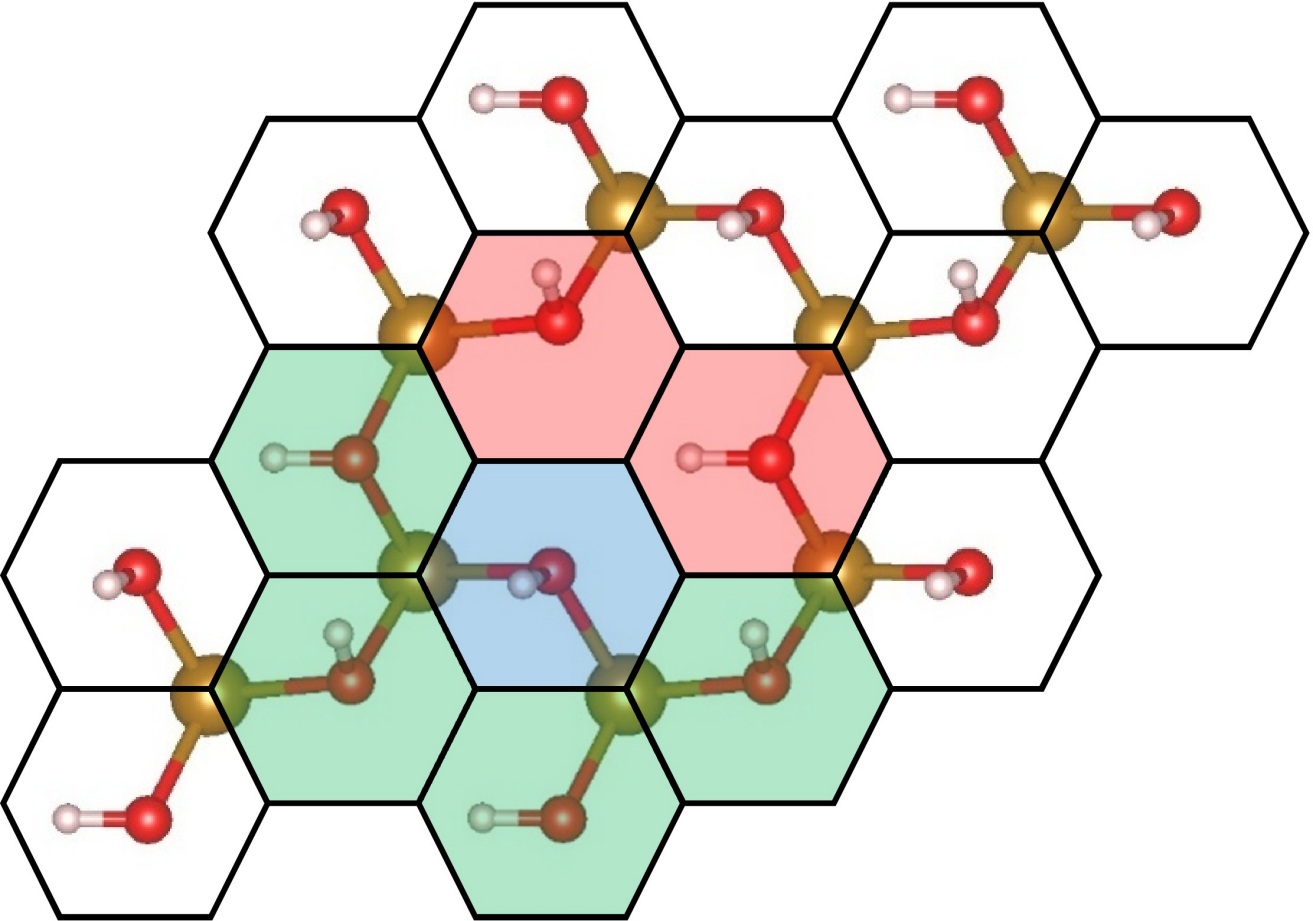
Hematite surface with a hexagonal grid. The blue site represents a chosen site, green sites are close neighbors that share an iron atom, and red sites are close neighbors that do not share an iron atom.

For the Monte Carlo simulation, we created a simulated n‐by‐n grid of hexagonally shaped active sites. Each surface intermediate created a hexagonal grid of sites, as shown in Figure 2. The active site is at the center of the hexagons, where a water molecule would adsorb on an oxygen vacancy site. In this work, we chose a 30‐by‐30 grid for all simulations.

Each active site has two bonds with neighboring iron atoms, as shown in Figure 2. Each iron atom has three bonds to active sites. In total, each active site (marked in blue) has six close neighbors, but only four of them (marked in green) share an iron atom with the site. Two sites (marked in red) do not share an iron atom with the active site, but were still considered first‐order neighbors in the calculation. Such neighboring relation is called an “interaction” in this paper, and we will show that such interactions affect the reaction energy. Under the “non‐interacting” sites model, reaction sites are unaffected by neighboring intermediates.

At each Monte Carlo iteration, a random site in the grid is chosen. The free energy change of the relevant catalysis reaction is calculated by taking the difference in the energy of the relaxed structure, gases, entropy, and zero‐point energy (ZPE) as used in other works[^10^, ^23^] and shown in equation (6) for reaction (3) as an example. The effect of pH on the free energy is calculated from the Nernst equation. The external bias is modeled as a constant electric field across the slab.

(6) $$\Delta G_{3}=E\left({}^{{}^{*}}O\right)-E\left({}^{{}^{*}}OH\right)+\frac{1}{2}E\left(H_{2}\right)+\Delta ZPE-T\Delta S-0.0592\cdot pH-eU$$

In this equation, E is the relaxed‐structure energy as calculated by DFT, ZPE is the zero‐point energy, T is the absolute temperature, S is entropy, and U is the external bias relative to the RHE. The energy of hydrogen represents the proton that leaves *OH in the reaction. Using Equation (6), we calculated the free energy changes at pH=13.6 for all five reactions, as shown in Table 1.

**Table 1:** Free energy change of single‐site reactions at pH=13 and no bias with respect to SHE. The sum of free energy changes is not 1.7 eV, as theory suggests, since DFT calculations give a water oxidation potential of 1.11 V and not the theoretical 1.23 V.

Reaction Number	ΔG at pH=13.6 and no bias w.r.t SHE [eV]
(1)	0.09
(2)	−0.84
(3)	1.05
(4)	0.84
(5)	0.08

The slab energy difference was calculated with regard to near‐neighbors as described for single site reactions and averaged over the six neighbors. The ZPE and entropy values were assumed to be unaffected by neighbor interactions. Then, the transition probability was calculated as follows:

(7) $$p=\left\{\begin{array}{cc} 1 & \Delta G\leq 0\\ e^{-\frac{\Delta G}{k_{B}T}} & \Delta G>0 \end{array}\right.$$

The simulation was run for a preset number of iterations. Each iteration result was saved in a MATLAB array.

Since the interaction between *OOH and *OH_2_ is a result of geometric relaxation, we assumed it has no kinetic barrier, so the transformation occurs automatically whenever *OOH and *OH_2_ sites are neighbors, regardless of the chosen site.

In addition to the effect of thermodynamics, kinetics is a key factor under working conditions. To measure the effect of incorporating kinetic barriers into the model, we considered the activation energy from Marcus,^[24]^ as shown in Equation (8) for Reactions (2)‐(5), which involve electron transfer.

(8) $$E_{a}=\frac{{\left(\Delta G+\lambda \right)}^{2}}{4\lambda }$$

where λ is the reorganization energy. We used a value of 1.5 eV from Graetzel's^[25]^ work on iron oxide, which involves the inner part of the reorganization energy. ΔG is calculated per reaction, as described earlier.

Since kinetic barriers involve an activation energy, we used Kinetic Monte Carlo (KMC) with the residence time algorithm (RTA)^[26]^ instead of the Metropolis algorithm. With RTA, we calculated reaction rates of all possible reactions on the grid by using Equation (9), where E_a,i_ is the activation energy of the reaction in the i′th site. Then we created a sequence of partial sums and define the sum of all N rates as R (Equation (10)). We assumed an identical k_0_ for all reactions, with a value of $k_{0}=\frac{k_{B}T}{h}\approx 6.2\cdot 10^{12}Hz$ , as was done by Rajan and Carter.[^27^, ^28^]

(9) $$k_{i}=k_{0}e^{-\frac{E_{a,i}}{k_{B}T}}$$

(10) $$R_{n}=\sum_{i=1}^{n}k_{i},\;\;R=R_{N}$$

(11) $$u∼U\left[0,1\right]$$

(12) $$R_{j-1}<uR\leq R_{j}$$

After calculating all sums, we draw a random number u from a uniform distribution and choose the reaction in site j, where j satisfies the term in Equation (12). The chosen reaction occurs with 100 % acceptance rate. For example, if the simulation has three sites with k_1_=2 Hz, k_2_=3 Hz, and k_3_=1 Hz, we get R_1_=2 Hz, R_2_=5 Hz, and R_3_=R=6 Hz. Then, to pick site 1, u needs to be between 0 and 1/3, a probability of $\frac{k_{1}}{R}=\frac{1}{3}$ . To pick site 2, u needs to be between 1/3 and 5/6, a probability of $\frac{k_{2}}{R}=\frac{1}{2}$ , and to pick site 3, u needs to be between 5/6 and 1, a probability of $\frac{k_{3}}{R}=\frac{1}{6}$ . In this way, the largest probability is to choose site 2, since it has the largest rate reflected in the largest range of u values between 1/3 and 5/6 Therefore, this method allows accounting for the relative rates of all sites.

## Results and Discussion

We will present the results of hematite surface coverage by reaction intermediates in two parts. First, we will present the results of Monte Carlo simulations without the effects of near‐neighbor interactions between the species on the active sites. Then, we will present the results with near‐neighbor interactions, especially the effect of the interaction between the two intermediates *OOH−*OH_2_.

## Without Neighbor Interactions

When sites do not interact with each other, the steady‐state configuration of the system can be calculated from transition probabilities:^[29]^

(10) $$\frac{\theta_{i}}{\theta_{j}}=\frac{P_{j\to j+1}}{P_{i\to i+1}}$$

where θ_i_ is the fraction of sites with the i'th species and $P_{i\to i+1}$ is the probability that a catalysis reaction would occur should a site with the i'th species be chosen. The magnitude of probabilities determines the number of iterations required to achieve a steady state.

Under standard operating conditions, T=298.15 K, pH=13.6, and no bias, all transition probabilities are very close to zero since the free energy difference of Reaction (3) is approximately 1 eV and $e^{-\frac{1eV}{298.15K•k_{B}}}\approx 10^{-17}$ . An increased bias is required to lower the free energy difference to initiate catalysis in the simulation and in the experiment.

**Figure 3:**
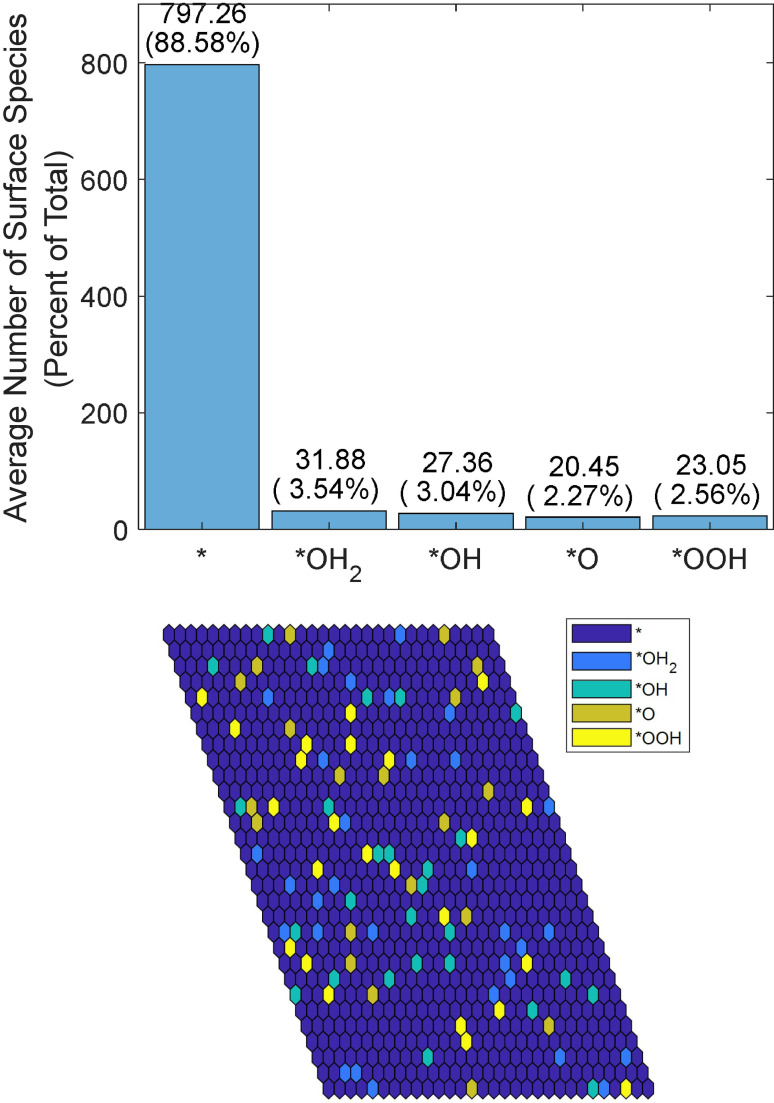
Number of intermediate species distributions without neighboring interactions averaged over 1000 Monte Carlo steps (top) and spatial distribution of intermediates (bottom). Simulations were run at pH=13.6 and a simulated voltage of 1.5 V.

Increasing the bias to 1 V lowers all free energy differences below zero except for Reaction (3), which has ΔG>1eV with no bias, and Reaction (1), which is independent of bias and pH. As a result, vacancies and *OH sites dominate the surface, while *OH_2_, *O, and *OOH sites have approximately equal coverages, as shown in Figure 3.

When the bias is increased beyond 1.06 V, all free energy differences except for reaction (1) become negative, so the ratio of vacancies to other species becomes approximately 30 : 1 : 1 : 1 : 1 since $\Delta G_{1}=0.0898$ and $e^{-\frac{0.0898\;eV}{298.15K•k_{B}}}\approx 0.03$ . A further increase in bias has no effect since the probabilities are unaffected when the free energy differences remain below zero. Such a high coverage of surface vacancies does not agree with the experimental results and may mean that neighboring interactions need to be considered.

## With Neighbor Interactions

When DFT was used to simulate the *OOH and *OH_2_ sites as near‐neighbors, they immediately transformed into *O and *OH sites, respectively. The transformation resulted from geometric relaxation of a slab with neighboring *OOH and *OH_2_ sites, which was unstable and resulted in a state with *O and *OH sites and a desorbed water molecule.

This interaction of species occurs due to two weak bonds: the O−H bond of *OH_2_ and the O−O bond of *OOH. The free energy change of the transition of *OH_2_ to *OH is close to zero with most neighbors when pH=0 and negative when pH=13.6, as shown in Table 2, which means it is almost energetically equivalent to that of *OH. The free energy change of the conversion of *O to *OOH is over 1 eV and the highest of all the reactions with some neighbors, which means that removing the extra OH is energetically favorable. Since *OH_2_ and *OOH are the largest species with three adsorbed atoms each, they can interact spatially more easily.

**Table 2:** Free energy differences (in eV) of all reactions with a near‐neighbor constant species. Numbers were calculated with T=298.15 K, pH=13.6, and no applied bias. (a) Reactions involving the *OOH−*OH_2_ neighbors that automatically result in *OH−*O neighbors after geometry optimization. (b) Reactions involving *OOH−*OH_2_ that will not occur since the interaction occurs before the reaction.

Constant species Reaction	*	*OH_2_	*OH	*O	*OOH
*→OH_2_	−0.072	−0.082	−0.007	−0.253	0.153^a^
*OH_2_→*OH	−0.690	−0.615	−0.982	0.339	−0.867^b^
*OH→*O	−0.183	−0.062	1.259	0.881	1.312
*O→*OOH	1.558	1.964^a^	0.758	1.189	0.544
*OOH→*	0.241	0.017^b^	0.194	−0.936	0.078

To show that neighboring species are possible, we calculated the formation energies of all 15 slabs. The formation energies of the five slabs with *OH are zero, since we take the *OH intermediate to be the “neutral” intermediate. All other formation energies are negative, except for the formation of *O−*OOH intermediates sharing an iron atom. However, the formation energy of the *O−*OOH slab is 0.106 eV, the lowest, in absolute values, of all formation energies, so the creation of *O and *OOH intermediates that share an iron atom is not impossible. All formation energies and calculation method are detailed in the Supporting Information and Table S3.

The addition of neighboring active site adsorbate interactions changes the simulation in two ways. The first is that the transition probabilities change according to the free energy changes detailed in Table 2, which may change the steady‐state coverage of the iron oxide surface. The second is the addition of the strong *OOH−*OH_2_ interaction that inhibits further reactivity for the participating species. The stability of the *OOH−*OH_2_ interaction becomes the only relevant reaction that affects the progress of water oxidation, since at increases simulated external bias, the free‐energy differences decrease or negative, making all transition probabilities closer and eventually equal to one, except for Reaction (1), which has bias‐independent free energy.

**Figure 4:**
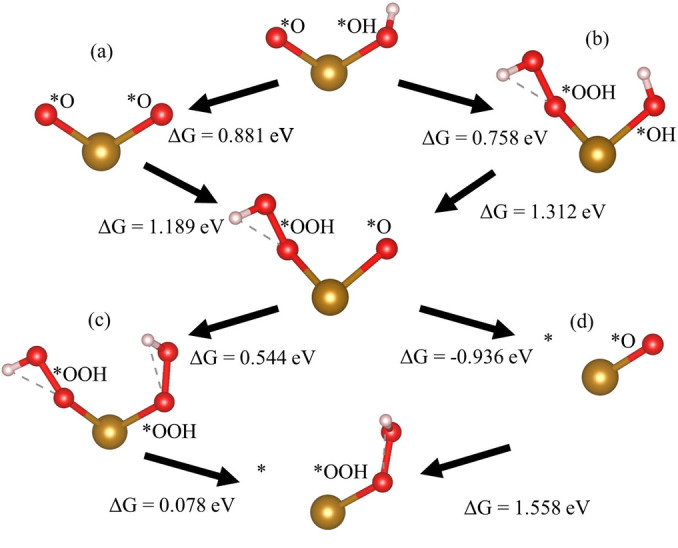
Different reaction paths from *OH−*O to *‐*OOH with pH=13.6. The four reaction paths are created by choosing one path between (a) and (b) and one between (c) and (d). The intermediates in each step are shown in labels. * alone denotes a vacant site.

The free energy differences in Table 2 reveal possible reaction paths in which neighboring species enable reactions at lower voltages. For example, when going from *O−*OH neighbors to *V−*OOH neighbors, there are several possible reaction paths, as shown in Figure 4. However, some paths undergo reactions with higher free‐energy differences, which increases the overpotential.

**Figure 5:**
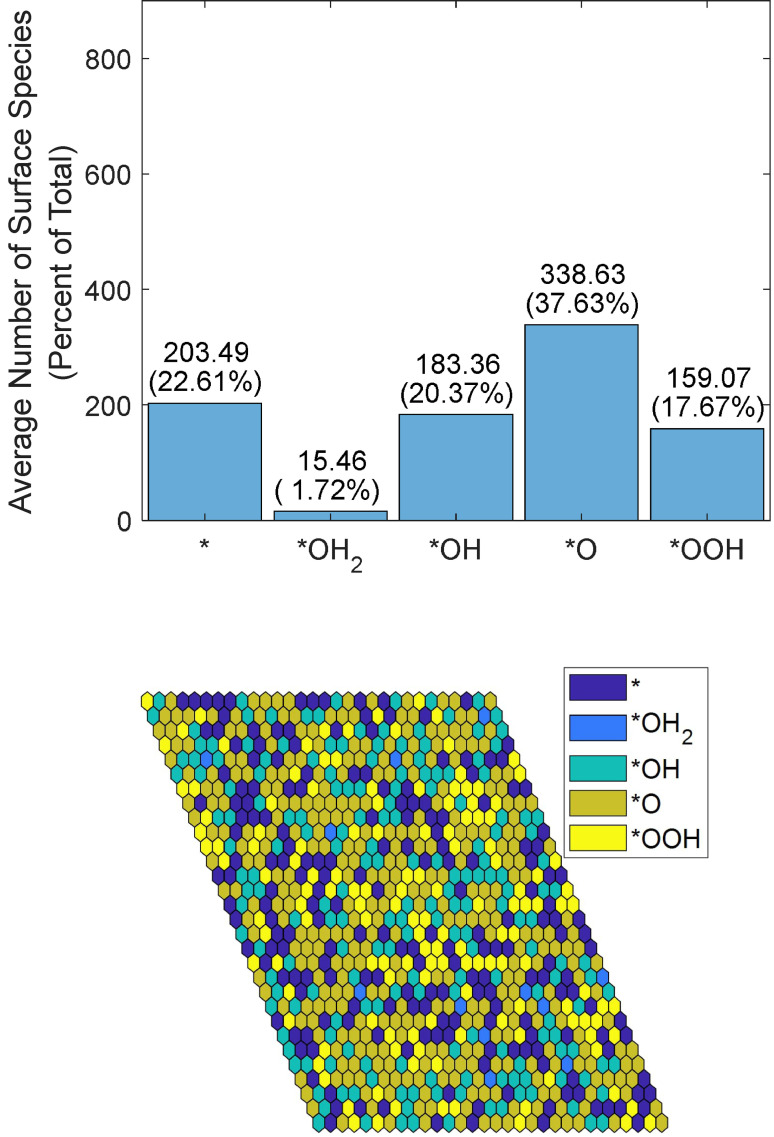
Average simulated distribution of species by number over 1000 frames (top). Spatial distribution of termination species with a simulated voltage of 3 V and pH=13.6 (bottom). Both simulations were run with 30×30 sites for 10,000 steps.

When the simulated applied voltage is higher than all free energy differences (exemplified by the extreme value of 3 V), all reaction probabilities go to one except for that of Reaction (1) next to *OH_2_. After several thousand cycles of Monte Carlo simulations, such as the one shown in Figure 5 at a steady state, there is a majority of *O intermediates.

The number of *OH and *OOH intermediates is almost equal and amounts to approximately half of the number of *O intermediates. According to the simulation results, *OH_2_ accounts for a small portion of the surface, and vacancies account for approximately 60 % of the number of *O intermediates. Overall, vacancies that are generated by the reaction cover approximately 23 % of the surface, *OH_2_ species cover approximately 2 %, *OH and *OOH species cover approximately 18 % each, and *O intermediates cover approximately 38 %. The surface coverage percentage fluctuates by approximately 1 % for each intermediate when averaged over 100 or more iterations at times, e. g., iterations 5000–5100 vs. iterations 5100–5200.

Simulations with an activation energy transition probability require a high input voltage to observe some reactions. For example, when the voltage lowers ΔG to zero, as shown in Equation (6), the activation energy changes according to Equations (8) to $E_{a}=\frac{\lambda }{4}=0.375\;eV$ . At room temperature, the exponent according to Equation (9) is approximately 10^−6^. Such value, compared to other rates with an exponent closer to unity, will create a rate of about six orders of magnitude smaller than other rates, so the reaction with such an activation energy will rarely happen.

**Figure 6:**
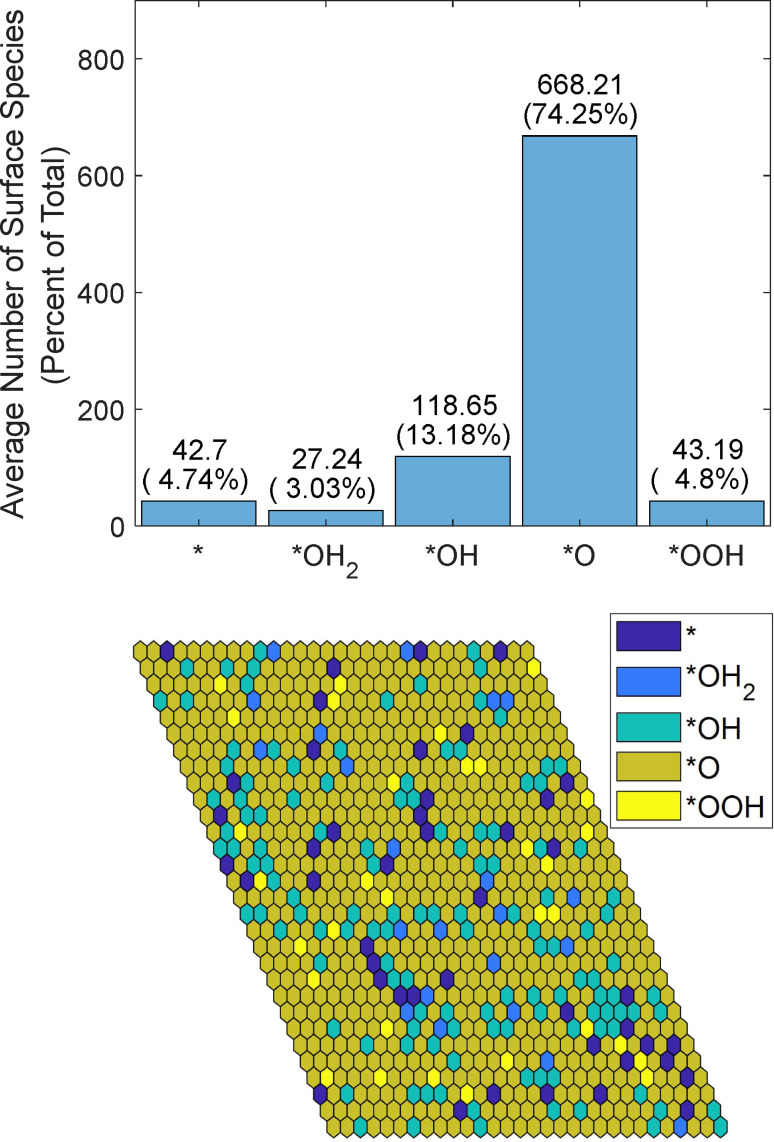
Average simulated distribution of species by number over 10000 frames (top). Spatial distribution of termination species with a simulated voltage of 2 V and pH=13.6 (bottom). Both simulations were run with 30×30 sites for 100,000 steps.

Raising the input voltage to 2 V lowers the barriers even more, so the only reactions with ΔG+λ>0 are the transitions from *OH and *O sites, which have approximately equal probabilities. Because of the *OOH−*OH_2_ interaction, *O becomes the dominant species on the surface, as shown in Figure 6.

When activation energy is used for transition probability, *O becomes even more dominant than when only thermodynamics is used. *OH has the next highest abundance, covering 13 % of the surface, and other species cover 3 %–5 % each. Applying an even higher voltage, one that reduces the sum of free energy change and reorganization energy below zero, produces the same results as the thermodynamic simulation.

In conclusion, we have shown a new possible explanation for the abundance of *O intermediates during OER catalysis by hematite. Previous experimental and theoretical work associated the dominance of a *O intermediate with a spectroscopic peak that remained apparent during the operation due to comparable surface absorption wavelengths and ab initio calculations for this specific *O intermediate.[^30^, ^31^] To do so, they used a single‐site model to show that the *O intermediate is dominant on the surface. In this work, we show that the single‐site approach may not be enough for a full description of catalysis since near‐neighbor interactions affect the surface coverage and that the source of the *O dominance is, in fact, the near‐neighbor interactions.

The mechanism of *O generation can be explained by the stability of the intermediates in the model accounting for near‐neighbor interactions. If there is proximity between two active sites holding *OH_2_ and *OOH interactions, they break down into *OH and *O intermediates and release a water molecule because both intermediates are unstable when they are near each other. The *O to *OOH reaction has a high free energy change, which makes the reverse reaction more favorable. In contrast, the *OH_2_ to *OH reaction has a negative free energy change, making this reaction favorable. Together, the two neighboring species are inclined to break down into new intermediates. Attractive forces between OH and H create a water molecule from the broken‐down species. The resulting intermediates (*OH and *O) have the highest free energy change for proceeding further in their reaction; thus, catalysis is hindered. The transition of *OH_2_ and *OOH intermediates to *OH and *O is found to be a reason for the domination of *O on the surface, and this result is in agreement with experimental measurements.[^11^, ^32^]

With this work, we demonstrated how neighboring sites may use each other's presence as neighbors to reduce the energy required for catalytic steps. To the best of our knowledge, this work is the first to show the effects of neighbor‐site species interactions that do not create a new intermediate. For water splitting with hematite, the interaction of neighbors appears to be an inherent problem, so the thermodynamic limit of the OER might be tighter than previously thought.

## Conflict of interest

The authors declare no conflict of interest.

1

## Supporting information

As a service to our authors and readers, this journal provides supporting information supplied by the authors. Such materials are peer reviewed and may be re‐organized for online delivery, but are not copy‐edited or typeset. Technical support issues arising from supporting information (other than missing files) should be addressed to the authors.

Supporting Information

Supporting Information

## Data Availability

The data that support the findings of this study are available in the supplementary material of this article.

## References

[1] J. Ren, S. Gao, H. Liang, S. Tan, L. Dong, in (Eds.: A. Scipioni, A. Manzardo, J. B. T.-H. E. Ren), Academic Press, **2017**, pp. 1–33.

[2] Y. Dou, L. Sun, J. Ren, L. Dong, in (Eds.: A. Scipioni, A. Manzardo, J. B. T.-H. E. Ren), Academic Press, **2017**, pp. 277–305.

[3] P. Sun , B. Young , A. Elgowainy , Z. Lu , M. Wang , B. Morelli , T. Hawkins , Environ. Sci. Technol. 2019, 53, 7103–7113. 31039312 10.1021/acs.est.8b06197

[4] S. J. A. Moniz , S. A. Shevlin , D. J. Martin , Z.-X. Guo , J. Tang , Energy Environ. Sci. 2015, 8, 731–759.

[5] M. Grätzel , Nature 2001, 414, 338–344. 11713540 10.1038/35104607

[6] A. Govind Rajan , J. M. P. Martirez , E. A. Carter , ACS Catal. 2020, 10, 11177–11234.

[7] T. A. Pham , Y. Ping , G. Galli , Nat. Mater. 2017, 16, 401–408. 28068314 10.1038/nmat4803

[8] H. Dotan , O. Kfir , E. Sharlin , O. Blank , M. Gross , I. Dumchin , G. Ankonina , A. Rothschild , Nat. Mater. 2013, 12, 158–164. 23142836 10.1038/nmat3477

[9] J. Rossmeisl , Z.-W. Qu , H. Zhu , G.-J. Kroes , J. K. Nørskov , J. Electroanal. Chem. 2007, 607, 83–89.

[10] P. Liao , J. A. Keith , E. A. Carter , J. Am. Chem. Soc. 2012, 134, 13296–13309. 22788792 10.1021/ja301567f

[11] N. Yatom , Y. Elbaz , S. Navon , M. Caspary Toroker , Phys. Chem. Chem. Phys. 2017, 19, 17278–17286. 28640300 10.1039/c7cp02404e

[12] C. A. Mesa , L. Francàs , K. R. Yang , P. Garrido-Barros , E. Pastor , Y. Ma , A. Kafizas , T. E. Rosser , M. T. Mayer , E. Reisner , M. Grätzel , V. S. Batista , J. R. Durrant , Nat. Chem. 2020, 12, 82–89. 31636394 10.1038/s41557-019-0347-1

[13] R. J. Lad , V. E. Henrich , Surf. Sci. 1988, 193, 81–93.

[14] G. Kresse , J. Furthmüller , Phys. Rev. B 1996, 54, 11169–11186. 10.1103/physrevb.54.11169 9984901

[15] G. Kresse , J. Furthmüller , Comput. Mater. Sci. 1996, 6, 15–50.

[16] G. Kresse , J. Hafner , Phys. Rev. B 1993, 47, 558–561. 10.1103/physrevb.47.558 10004490

[17] G. Kresse , J. Hafner , Phys. Rev. B 1994, 49, 14251–14269. 10.1103/physrevb.49.14251 10010505

[18] S. L. Dudarev , G. A. Botton , S. Y. Savrasov , C. J. Humphreys , a. P. Sutton , Phys. Rev. B 1998, 57, 1505–1509.

[19] N. J. Mosey , P. Liao , E. A. Carter , J. Chem. Phys. 2008, 129, 14103. 10.1063/1.2943142 18624466

[20] P. E. Blöchl , O. Jepsen , O. K. Andersen , Phys. Rev. B 1994, 49, 16223–16233. 10.1103/physrevb.49.16223 10010769

[21] G. Lehmann , M. Taut , Phys. Status Solidi 1972, 54, 469–477.

[22] J. Nocedal, S. Wright, *Numerical Optimization*, Springer, **2017**.

[23] N. Yatom , O. Neufeld , M. Caspary Toroker , J. Phys. Chem. C 2015, 119, 24789–24795.

[24] R. A. Marcus , N. Sutin , Biochim. Biophys. Acta Rev. Bioenerg. 1985, 811, 265–322.

[25] M. Graetzel, *Heterogeneous Photochemical Electron Transfer*, CRC Press, Boca Raton, Fla., **1989**.

[26] A. B. Bortz , M. H. Kalos , J. L. Lebowitz , J. Comput. Phys. 1975, 17, 10–18.

[27] A. Govind Rajan , E. A. Carter , Energy Environ. Sci. 2020, 13, 4962–4976.

[28] J. Huang , M. Li , M. J. Eslamibidgoli , M. Eikerling , A. Groß , JACS Au 2021, 1, 1752–1765. 34723278 10.1021/jacsau.1c00315 PMC8549051

[29] M. Andersen , C. Panosetti , K. Reuter , Front. Chem. 2019, 7, 202. 31024891 10.3389/fchem.2019.00202 PMC6465329

[30] O. Zandi , T. W. Hamann , Nat. Chem. 2016, 8, 778. 27442283 10.1038/nchem.2557

[31] B. Klahr , T. Hamann , J. Phys. Chem. C 2014, 118, 10393–10399.

[32] N. Snir , M. C. Toroker , J. Chem. Theory Comput. 2020, 16, 4857–4864. 32603108 10.1021/acs.jctc.9b00595

