# Supplementary material for: Simulations to Cover the Waterfront for Iron Oxide Catalysis
Source: Chemphyschem. 2022 Feb 15;23(6):e202200025. doi: 10.1002/cphc.202200025 (PMC9303966; doi:10.1002/cphc.202200025)
Supplement: Supplementary file 1 — Supporting Information [file CPHC-23-0-s002.pdf]

ChemPhysChem

Supporting Information

Simulations to Cover the Waterfront for Iron Oxide Catalysis

Nadav Snir and Maytal Caspary Toroker*

Supporting Information

Table S1 – reaction free energies for pH = 0, V = 0, T = 298.15K with near-neighbor constant species. (a) Reactions involving the *OOH-*OH2 neighbors which automatically end in *OH-*O neighbors after geometry optimization. (b) Reactions involving the *OOH-*OH_2 unable to occur due to species interaction causing instability (high energy) of this reaction intermediate.

Constant species / Reaction	*	*OH_2	*OH	*O	*OOH
* → OH_2	-0.072	-0.082	-0.007	-0.253	0.153[a]
*OH_2 → *OH	0.115	0.190	-0.177	1.144	-0.062[b]
*OH → *O	0.988	0.743	2.064	1.686	2.117
*O → *OOH	2.363	2.769[a]	1.563	1.994	1.349
*OOH → *	1.046	0.822[b]	0.999	-0.131	0.883

Choosing Which Neighbors to Include

We tested two slabs, one with two *O intermediates that have a shared iron atom and one with two *O intermediates at the same distance, but without a shared iron atom, as shown in figure S1:

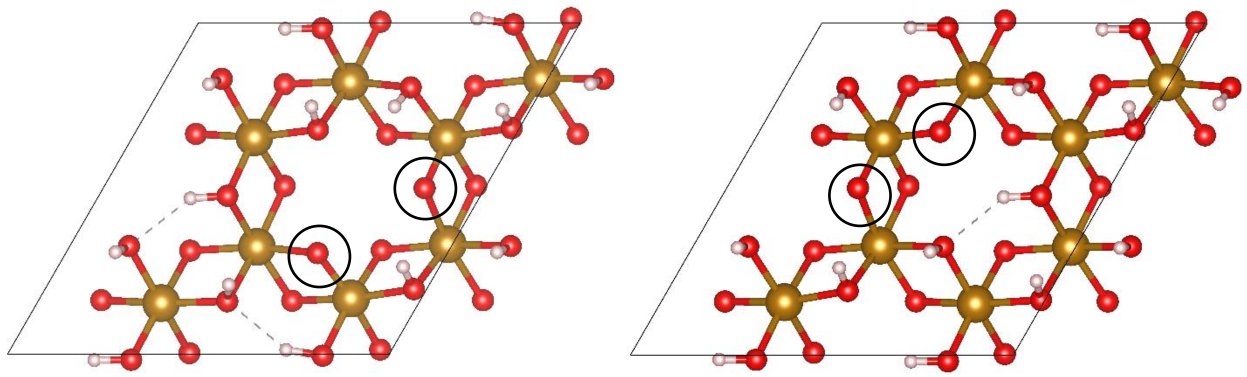

Figure S1 – two slabs with *O-*O interaction. (left) Without a shared iron atom. (right) With a shared iron atom.

The two slabs had a total energy difference of 0.176 eV, which amounts to a difference of 0.088 eV for the entire reaction. Due to the negligible difference, we decided to include all six neighbors in our calculations.

Calculating the Reaction Energy With Multiple Neighbors

Each slab in the calculation contained two sites of interest: the reaction site and one first-order neighbor. All other sites contained *OH terminations. In order to calculate an energy difference with multiple neighbors, we tested slabs of the *OH → *O reaction with an increasing number of *O neighbor sites.

Table S2 – energy difference between active intermediates of the *OH → *O reaction with a different number of *O neighbors.

Number of *O neighbors	Site energy change ΔE (eV)
1	5.47
2	5.62
3	5.70
4	5.73

The results shown in Table S2 are not linear, but show a trend. Therefore, we decided to average the energy differences of the six neighbors to avoid the need to calculate hundreds of slabs with different species combinations. The main patterns of the simulation do not change significantly with small differences in site energy.

Adding a Water Molecule to *OOH-*OH_2 Slab

When adding a water molecule to *OOH-*OH_2 slab, the molecule stabilizes the slab and prevents the deprotonation of adsorbed *OH_2. However, if the OH^- from *OOH is closer to *OH_2 than the water molecule, deprotonation occurs, as shown in Figure S2.

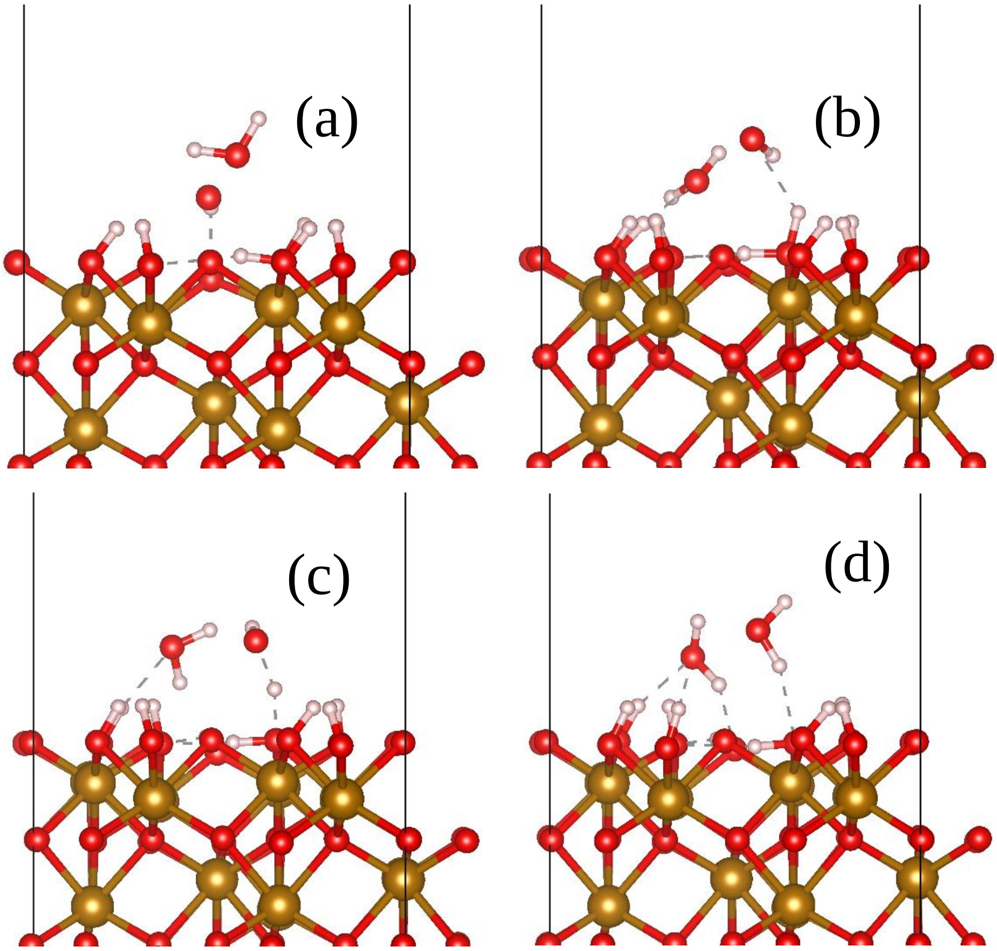

Figure S2 – position of OH^- and water molecule. (a) original location – water near $*OH_2$ and OH^- near *OOH. (b) manually exchanging positions of OH^- and water molecule. (c) deprotonation of adsorbed $*OH_2$. (d) final state – two water molecules.

While the water molecule stabilizes the adsorbed $*OH_2$, if it moves and an OH^- ion takes its place, deprotonation still occurs. Furthermore, the final step (d) of Figure S2, with two desorbed water molecules, is still lower in energy than one desorbed water molecule and adsorbed *OOH and $*OH_2$ neighbors.

Formation Energies of Slabs With Neighboring Intermediates

To calculate the formation energies of iron atoms with two intermediates, we calculated the theoretical energy of a slab with two intermediates not sharing an iron atom. Then, we subtracted that energy from the energy of a slab with intermediates sharing an iron atom.

To calculate the theoretical energy of a slab with two intermediates, we added the formation energy of *OH-*X slabs from all *OH slabs with the following formula:

$$E_{*X_1-*X_2,non-shared} = E_{*OH-*X_1} - E_{*OH-*OH} + E_{*OH-*X_2} - E_{*OH-*OH} + E_{*OH-*OH}$$
$$E_{*X_1-*X_2,non-shared} = E_{*OH-*X_1} + E_{*OH-*X_2} - E_{*OH-*OH}$$

With that energy difference, we can calculate the formation energy of the slabs with intermediates that share an iron atom:

$$\Delta E_f = E_{*X_1-*X_2,shared} - E_{*X_1-*X_2,non-shared}$$

From the formation energy equation, it immediately follows that all *X-*OH intermediates have a formation energy of zero (since they already share an iron atom with *OH). Table S3 details the formation energies of all 15 slabs.

Table S3 – formation energies of slabs with a shared iron atom from slabs without a shared iron atom, in eV. Bottom values were omitted for symmetry reasons.

Intermediate 2 / Intermediate 1	*	$*OH_2$	*OH	*O	*OOH
*	-0.455	-0.584	0	-2.152	-0.552
$*OH_2$	-	-0.733	0	-2.6427	-0.231
*OH	-	-	0	0	0
*O	-	-	-	-0.757	0.107
*OOH	-	-	-	-	-0.320

Example INCAR for geometric relaxation:

```
SYSTEM= feouj0       # title of the task

# Dimensions of arrays

EMIN =  -20        #minimum energy for evaluation of DOS

EMAX =  20            #maximum energy for evaluation of DOS

NEDOS=  20000         #number of grid points in DOS

# Start parameter for this run:

ISTART = 1           # job   : 0-new  1-cont  2-samecut # changed
from 1

ICHARG = 1          # charge: 1-file 2-atom 10-const

#INIWAV = 1          # electr: 0-lowe 1-rand  2-diag

PREC = Accurate      # medium, high low

ALGO = Very_Fast          #Normal,VeryFast...#specify the electronic
minimisation algorithm

#TIME= 0.4           #controls the trial time step for IALGO=5X, for
the initial (steepest descent) phase of IALGO=4X

# DOS related values:

ISMEAR = -5          #-5,-4,-3,-2,0,N #0-Gaussian smearing

                     #determines how the partial occupancies
f(n,k)are set for each orbital

SIGMA = 0          #broadening in eV -4-tet -1-fermi 0-gaus

EDIFF=1E-4           #global break condition for the electronic SC-
loop

NELM=100             #maximum number of electronic SC
(selfconsistency) steps

NELMIN = 6

LWAVE=.TRUE.         #These tags determine whether the orbitals
(file WAVECAR),

LCHARG=.TRUE.        #the charge densities (file CHGCAR and CHG) are
written

# Spin and symmetry parameters

MAGMOM= 62*0 4*4.2 4*-4.2 4*4.2 4*-4.2 4*4.2 4*-4.2 4*4.2 4*-4.2
24*0  #Specifies the initial magnetic moment for each atom (order of
POSCAR)

ISPIN=2             #1=non/2= spin polarized calculations are
performed
```

```
ISYM=0              #-1,0,1,2,3 #switch symmetry on (ISYM=1, 2 or 3)
or off (ISYM=-1 or 0)

LMAXMIX=4           #controls up to which l quantum number the
onsite PAW charge densities are passed through the charge density
mixer and written to the CHGCAR file

# Ionic relaxation

EDIFFG = -3.0E-2    #Def=EDIFF*10 defines the break condition for
the ionic relaxation loop

LORBIT=10           #11=>RWIGS in INCAR is not read. DOSCAR and lm
decomposed PROCAR file

NSW = 1000            #maximum number of ionic steps

IBRION = 2          #=-1 for NSW=0 or NSW=1 #determines how the ions
are updated and moved

#IOPT = 7

POTIM = 0.25        #For IBRION=1,2 or 3, POTIM serves as a scaling
constant for the forces

ISIF=0              #0-6 =0 if IBRION=0(MD)else =2 #controls whether
the stress tensor is calculated

#ADDGRID = .TRUE.

LREAL=.False.       #projection done in: FALSE=reciprocal/TRUE=real
space

#LREAL = Auto         # Speed things up

#NSIM=1              #if spacified the RMM-DIIS
algorithm(IALGO=48)works in a blocked mode

NPAR=4              #~sqrt(number of cores) or number of cores per
compute node

KPAR=4              #KPAR is the number of k-points that are to be
treated in parallel

#NBAND              #Def: NELECT/2 + NIONS/2 (non-spinpolarized)

                    #     0.6*NELECT + NMAG (spin-polarized)

                    #determines the actual number of bands in the
calculation

ENCUT = 700 eV      #Cut-off energy for plane wave basis set in eV.
largest ENMAX from POTCAR-fil

#On site Coulomb interaction: L(S)DA+U

LDAU=.TRUE.         #TRUE-switches on the L(S)DA+U
```

LDAUTYPE= 2 #specifies which type of L(S)DA+U approach will be used

LDAUPRINT= 0 #0,1,2. controls what to write in OUTCAR

LDAUL= -1 2 -1 #-1=no on-site terms added, 1= p, 2= d, 3= f

LDAUU= 0.0 4.6 0.0 #specifies the effective on-site Coulomb interaction parameters

LDAUJ= 0.0 0.3 0.0 #specifies the effective on-site Exchange interaction parameters

MATLAB Code

The following code uses values calculated by VASP to run a Monte Carlo simulation of surface reactions of water splitting by hematite.

Required input for "ExpectationValue" functions (depending on which simulation to run) is number of unit cells (with 3 sites in each cell), K is the number of steps, pH, T is absolute temperature, V is external bias, Epsilon is convergence threshold (for convergence analysis only. For regular usage, use 1e-4), and Sk is an alternative initial frame (otherwise, the default initial frame is *OH on all sites).

The outputs are HExp, an expectation value of surface energy (not used in this paper), Sk, the last frame (for continuation jobs), and MovieFile, a 3D matrix containing all simulation steps.

The analysis and animation functions, DrawHexagons, AverageManyFrames, CreateHexMovie, and SingleHexMovieFrame use the MovieFile variable.

EnergyMatrix is a symmetric matrix comprised of slab energies of *A-*B, where *A and *B are the different termination species. The energies are given in the POSCAR file list in this SI. The order of the rows and columns is 1) * 2) *OH_2 3) *OH 4) *O 5) *OOH.

For example, the energy in the 4th row, 5th column (or 5th row, 4th column) is for the *O-*OOH slab.

With Neighbors:

```
% Run simulation with neighbor interactions

function [HExp, Sk, MovieFile] =
ExpectationValuesSurfaceWithNeighborsMetropolis(UnitCells
, K, pH, T, V, Epsilon, Sk)

% Load energy matrix (energy table of pairs of
terminations)
EnergyMatrix = 0;
load('EnergyMatrix.mat','EnergyMatrix');
EnergyMatrix = EnergyMatrix / 2;

n = 3*UnitCells;
if ~exist('Sk','var')
    Sk = 3*ones(n);
end
```

```
% Inititate step matrix
MovieFile = zeros(n,n,K);

kB = 1.38e-23 / 1.6e-19; % Boltzmann constant in eV/K

HTot = zeros(1,K);
PrevH = CalculateSurfaceEnergyNearNeig(Sk,
EnergyMatrix,true);
% PrevH = CalculateSurfaceEnergy(Sk, Energies);
for i = 1:K

    % Calculate energy of current state
    [HTot(i), Sk, NWk] =
CalculateSurfaceEnergyNearNeig(Sk, EnergyMatrix,true);

    % Pick random site
    NewCoords = [randi([1 n]) randi([1 n])];
    Skp = Sk;
    ReactionNum = Skp(NewCoords(1),NewCoords(2));
    Skp(NewCoords(1),NewCoords(2)) =
mod(Skp(NewCoords(1),NewCoords(2)),5) + 1;
    OldSkp = Skp;

    % Calculate energy with the chosen site's reaction
    [NewH, Skp, NWkp] =
CalculateSurfaceEnergyNearNeig(Skp, EnergyMatrix,false);
    dE = NewH - HTot(i);

    % Calculate dG - free energy change
    dG = dE + DeltaGExtras(ReactionNum,pH,T,V, NWkp -
NWk);

    % Decide whether to accept or reject the step
    if dG < 0 || rand(1) < exp(-dG/(kB * T))
        Sk = Skp;
    end
    PrevH = HTot(i);
    MovieFile(:,:,i) = Sk;

end

Ediff = diff(HTot/n^2);
LastEps = find(Ediff > Epsilon,1,'last');
HExp = mean(HTot(LastEps+1:end));

end
```

With activation energy:

```
% Run simulation with activation energy and constant
lambda

function [HExp, Sk, MovieFile] =
ExpectationValuesSurfaceWithNeighborsAEConstantLambda(Uni
tCells, K, pH, T, V, Epsilon, Sk)

% Load energy matrix (energy table of pairs of
terminations)
EnergyMatrix = 0;
load('EnergyMatrix.mat','EnergyMatrix');
EnergyMatrix = EnergyMatrix / 2;
Lambda = 1.5; % eV, from Graetzel
n = 3*UnitCells;
if ~exist('Sk','var')
    Sk = 3*ones(n);
end

% Inititate step matrix
MovieFile = zeros(n,n,K);

kB = 1.38e-23 / 1.6e-19; % Boltzmann constant in eV/K

HTot = zeros(1,K);
PrevH = CalculateSurfaceEnergyNearNeig(Sk,
EnergyMatrix,true);

for i = 1:K

    % Correct *OOH-*OH2 interactions before calculating
activation energies
    % to save time.
    [~, Sk, ~] = CalculateSurfaceEnergyNearNeig(Sk,
EnergyMatrix,true);

    % Create a matrix of all activation energy exponents
(assuming a
    % uniform rate coefficient). Don't correct for OOH-
OH2 neighbors
    [AEMat, IndMat, Sk] = ActEnMat(Sk, EnergyMatrix, pH,
T, V, Lambda);

    % Convert the matrix to a vector of probabilities

    AEMat = AEMat(:);
```

```
    ProbVector = cumsum(AEMat / sum(AEMat));

    % Draw a uniformly distributed random number
    P = rand;

    % Find the site where the probability lies
    ChosenSite = find(ProbVector >= P,1);

    % Convert the site number to matrix indices
    [NR, NC] = ind2sub([n, n],IndMat(ChosenSite));

    % Advance matrix by one
    Sk(NR, NC) = mod(Sk(NR,NC),5) + 1;

    PrevH = HTot(i);
    MovieFile(:,:,i) = Sk;

end

Ediff = diff(HTot/n^2);
LastEps = find(Ediff > Epsilon,1,'last');
HExp = mean(HTot(LastEps+1:end));

end

function [AEMat, IndMat CurrentState] =
ActEnMat(CurrentState, EnergyMatrix, pH, T, V, Lambda)

n = size(CurrentState,1);
AEMat = zeros(size(CurrentState));
kB = 1.38e-23 / 1.6e-19; % Boltzmann constant in eV/K

IndMat = 1:n^2;
m = 1;
for k = IndMat
    [i, j] = ind2sub([n, n],k);
    CurrentEnergy = NeighborEnergy(CurrentState,
EnergyMatrix, [i, j]);
    ReactionNum = CurrentState(i,j);
    NewState = CurrentState;
    NewState(i,j) = mod(NewState(i,j),5) + 1;

    NewEnergy = NeighborEnergy(NewState, EnergyMatrix,
[i, j]);

    dE = NewEnergy - CurrentEnergy;

    % Calculate dG and activation energy from Marcus
```

```
    dG = dE + DeltaGExtras(ReactionNum,pH,T,V, 0);
    Ea = dG;
    if ReactionNum > 1 && dG + Lambda > 0
        Ea = (dG + Lambda)^2 / (4*Lambda);
    end
    if Ea < 0
        Ea = 0;
    end

    AEMat(m) = exp(-Ea/(kB*T));
    m = m + 1;
end

end


function NeigEnergy = NeighborEnergy(Sk, EnergyMatrix,
Location)

EnergyMatrix = EnergyMatrix - min(EnergyMatrix(:)); % Set
lowest energy to zero

NewSk = ExtendSurface(Sk);

% Take only a 3x3 square around the chosen site, since
nothing else changes
% or matters
LocalMatrix =
NewSk(Location(1):Location(1)+2,Location(2):Location(2)+2
);

LocalNeigMat = [0 1 1; 1 0 1; 1 1 0];

PowerMatrix = 10.^LocalMatrix;

% Convolve the power matrix with the neighbor matrix and
take only the
% central value, the one which has the chosen site
ConvNeighborMat = conv2(PowerMatrix,LocalNeigMat,'valid')
+ LocalMatrix(2,2);

% Convert the number of neighbors to a vector in a
resource-friendly matter
PowerSeries = 10.^(5:-1:0);
CurrentEnergy =
floor(mod(ConvNeighborMat./PowerSeries,10));

% Calculate the total energy of the state
```

```
TotalEnergies = zeros(5);
TotalEnergies(CurrentEnergy(6),:) = CurrentEnergy(5:-
1:1);
TotalEnergies = TotalEnergies * EnergyMatrix / 6;

NeigEnergy = sum(diag(TotalEnergies));

end
```

With neighbors, with and without activation energy:

```
% Calculate energy with neighbor interactions

function [HTot, InnerSk, NewWaters] =
CalculateSurfaceEnergyNearNeig(Sk,EnergyMatrix,Fix)

EnergyMatrix = EnergyMatrix - min(EnergyMatrix(:)); % Set
lowest energy to zero

% Include OOH-OH2 interactions?
if Fix
    [NewSk, ~, NewWaters] = FixOOHOHH(Sk);
else
    NewSk = ExtendSurface(Sk);
    NewWaters = 0;
end

% Create neighbor matrix
NeigMat = GetNeighbors(Sk);

% Take only inner square (the original one) to help
calculate neighbors
% later
InnerSk = NewSk(2:end-1,2:end-1);

% Create a matrix of 10 to the power of termination
number to differentiate
PowerMatrix = 10.^NewSk;

% Convolve all locations with the three neighbor
matrices. Add InnerSk so
% the units digit will tell the original species

% LocalNeighbors includes all six neighbors
```

```
Conv1 = conv2(PowerMatrix,LocalNeighbors(1,true),'valid')
+ InnerSk;


% All six neighbors are considered, no need to add
convolutions
TotalConv = Conv1;

% Convert all numbers to a series of integer
representations
TotalConv = num2str(TotalConv(:),'%06i');
TotalConv = TotalConv - '0';
TotalEnergies = zeros(5);

% Go over all sites
for i = 1:size(TotalConv,1)
    % Get current species (and location on TotalEnergies)
    CurSpec = TotalConv(i,6);

    % Site numbers to total energies (goes from 5 to 1
because the powers
    % of 10 make the 10^5 digit the first one)
    TotalEnergies(CurSpec,:) = TotalEnergies(CurSpec,:) +
TotalConv(i,5:-1:1);
end

% Divide by twelve since there are SIX neighbors
TotalEnergies = TotalEnergies * EnergyMatrix / 12;

% The sum of all energies is the sum of the DIAGONAL of
the TotalEnergies
% matrix
HTot = sum(diag(TotalEnergies));

end

% Extend current surface to include periodic images of
current surface
function ExtendedSurface = ExtendSurface(OriginalSurface)

% Assume square
SurfSize = size(OriginalSurface,1);

MultSurf = repmat(OriginalSurface,3,3);

ExtendedSurface =
MultSurf(SurfSize:2*SurfSize+1,SurfSize:2*SurfSize+1);
```

```
end

function NeigMat = GetNeighbors(InputMatrix)

% Get matrix size (assume square)
MatSize = size(InputMatrix,1);

% Create matrices for calculation of neighbor matrix
[H, V] = meshgrid(1:MatSize);

% Add both matrices, subtract 2 to start from 0,
% find mod 3 and add 1 to assign numbers from 1 to 3 only
NeigMat = flipud(mod(H + V - 2,3) + 1);

end

% Create matrix of neighbors
function LocalNeigMat = LocalNeighbors(NeighborNum,
Rotated)

if ~exist('Rotated','var')
    Rotated = 0;
else
    Rotated = 2;
end


LocalNeigMat = [0 1 1; 1 0 1; 1 1 0];
% For some reason, the convolution rotates the B matrix
180 degrees.
% This should fix it.
LocalNeigMat = rot90(LocalNeigMat,Rotated);

end

% Find *OOH and *OH2 neighbors and convert them to *O and
*OH, respectively
function [AdjustedSurface, NeigMat, NewWaters] =
FixOOHOHH(OriginalMatrix)

[RLoc, CLoc] = find(OriginalMatrix == 5);
AdjustedSurface = ExtendSurface(OriginalMatrix);
NeigMat = ExtendSurface(GetNeighbors(OriginalMatrix));

% Default value
NewWaters = 0;

for i = 1:numel(RLoc)
```

```
    % Get coordinates in EXTENDED matrices
    CurR = RLoc(i) + 1;
    CurC = CLoc(i) + 1;

    % Neighbor type
    CurNeig = NeigMat(CurR, CurC);

    % Nearest neighbors of current OOH
    LocalMat = AdjustedSurface(CurR-1:CurR+1,CurC-
1:CurC+1);

    % Multiply possible OHH's and neighbor matrix to see
if any neighbors
    % are available
    OHHMat = (LocalMat == 2) .* LocalNeighbors(CurNeig);

    % Find first neighbor (only one changes location)
    [RVal, CVal] = find(OHHMat,1,'first');

    % If there is an OHH termination next to OOH, change
the OOH to O and
    % OHH to OH
    if RVal
        % Change *OOH to *O and *OHH to *OH
        AdjustedSurface = AdjustSurface(AdjustedSurface,
CurR, RVal, CurC, CVal);
        % Takes care of edge positioned *OHH or *OOH that
affect results
        % after fixing one intermediate
        AdjustedSurface =
ExtendSurface(AdjustedSurface(2:end-1,2:end-1));
        % Increase the number of water molecules that
were released
        % (unnecessary right now)
        NewWaters = NewWaters + 1;
        % fprintf('DING\n');
    end
end

% Recreate extended surface (because the loop goes over
the inner square
% only and ignores edges)
AdjustedSurface = ExtendSurface(AdjustedSurface(2:end-
1,2:end-1));

end
```

```
% Change *OOH to *O and *OH2 to *OH where *OOH and *OH2
are neighbors
function AdjustedSurface = AdjustSurface(OriginalSurface,
CurR, RVal, CurC, CVal)

SurfSize = size(OriginalSurface);

AdjustedSurface = OriginalSurface;

% Change *OHH to *OH
AdjustedSurface(CurR + RVal - 2, CurC + CVal - 2) = 3;

% Change *OOH to *O
AdjustedSurface(CurR, CurC) = 4;

% If the change is on the first row (extension), also
change the next to
% last.
if CurR + RVal - 2 == 1
    AdjustedSurface(SurfSize(1) - 1, CurC + CVal - 2) =
3;
end

% If the change is on the last row (extension), also
change the second
% (original).
if CurR + RVal - 2 == SurfSize(1)
    AdjustedSurface(2, CurC + CVal - 2) = 3;
end

% If the change is on the first colum (extension), also
change the next to
% last.
if CurC + CVal - 2 == 1
    AdjustedSurface(CurR + RVal - 2, SurfSize(2) - 1) =
3;
end

% If the change is on the last column (extension), also
change the second
% (original).
if CurC + CVal - 2 == SurfSize(2)
    AdjustedSurface(CurR + RVal - 2, 2) = 3;
end

end
```

No Neighbors:

```
% Run simulation with no neighbor interaction

function [HExp, Sk, MovieFile] =
ExpectationValuesSurfaceNoNeighborsMetropolis(UnitCells,
K, pH, T, V, Epsilon)

Energies = [-226.9741   -256.7845   -249.3570   -238.0812
-257.4178]/2; % From [1]

n = 3*UnitCells;

MovieFile = zeros(n,n,K);

Sk = 3*ones(n);

kB = 1.38e-23 / 1.6e-19; % Boltzmann constant in eV/K

HTot = zeros(1,K);
PrevH = CalculateSurfaceEnergy(Sk, Energies);
for i = 1:K

    % Calculate energy of current state
    HTot(i) = CalculateSurfaceEnergy(Sk, Energies);

    % Pick random site
    NewCoords = [randi([1 n]) randi([1 n])];
    Skp = Sk;
    ReactionNum = Skp(NewCoords(1),NewCoords(2));
    Skp(NewCoords(1),NewCoords(2)) =
mod(Skp(NewCoords(1),NewCoords(2)),5) + 1;

    % Calculate energy with the chosen site's reaction
    NewH = CalculateSurfaceEnergy(Skp, Energies);
    dE = NewH - HTot(i);

    % Calculate dG and decide whether to accept or reject
the step
    dG = dE + DeltaGExtras(ReactionNum,pH,T,V);
    if dG < 0 || rand(1) < exp(-dG/(kB * T))
        Sk = Skp;
    end
    PrevH = HTot(i);

    MovieFile(:,:,i) = Sk;
end
```

```
Ediff = diff(HTot/n^2);
LastEps = find(Ediff > Epsilon,1,'last');
HExp = mean(HTot(LastEps+1:end));

end


% Calculate surface energy with no neighbor interaction

function [HTot] =
CalculateSurfaceEnergy(Sk,StateEnergies)

StateEnergies = StateEnergies - min(StateEnergies); % Set
lowest energy to zero

NewSk = zeros(size(Sk));

% Assign energy to each state
for i = 1:max(Sk(:))
    NewSk(Sk == i) = StateEnergies(i);
end

% NewSk = AddInfinity(Sk,NewSk);
% Sum all energies
HTot = sum(NewSk(:));

end
```

All simulations:

```
% Add entropy, ZPE, and other reactants' energies to
calculate dG from dE

function [dG] = DeltaGExtras(ReactionNumber, pH, T, V,
Waters)

if ~exist('T','var')
    T = 298.15; % default temperature in K
end

if ~exist('pH','var')
    pH = 0; % default pH
end

if ~exist('V','var')
    V = 0; % default voltage
```

```
end

if ~exist('Waters','var')
    Waters = 0; % no extra water molecules
end

[ZPE, S, E] = GetParams;
R = 8.314; % Universal gas constant (in J/mol*K)
F = 1.6e-19 * 6.022e23; % Nernst constant in C/mol

switch ReactionNumber
    case 1 % V -> OHH
        dG = -E.H2O + ZPE.OHH - ZPE.H2O - ZPE.V -
T*(S.OHH - S.H2O);
    case 2 % OHH -> OH
        dG = E.H2/2 + ZPE.OH + ZPE.H2/2 - ZPE.OHH -
T*(S.OH + S.H2/2 - S.OHH) - V - R*T/F*log(10)*pH;
    case 3 % OH -> O
        dG = E.H2/2 + ZPE.O + ZPE.H2/2 - ZPE.OH - T*(S.O
+ S.H2/2 - S.OH) - V - R*T/F*log(10)*pH;
    case 4 % O -> OOH
        dG = E.H2/2 - E.H2O + ZPE.OOH + ZPE.H2/2 - ZPE.O
- ZPE.H2O - T*(S.OOH + S.H2/2 - S.O - S.H2O) - V -
R*T/F*log(10)*pH;
    case 5 % OOH -> V
        dG = E.O2 + E.H2/2 + ZPE.H2/2 + ZPE.O2 + ZPE.V -
ZPE.OOH - T*(S.O2 + S.H2/2 + S.V - S.OOH) - V -
R*T/F*log(10)*pH;
end

% If the *OOH + *OHH -> *OH + *O + H2O change has
occured, the reactions
% are different than regular
if Waters > 0
    if ReactionNumber == 4
        % *O -> *OOH and then *OOH + *OHH -> *O + *OH +
H2O (water is added
        % later). Total is *O + *OHH -> *O + *OH + H
        dG = ZPE.OH + ZPE.H2/2 - ZPE.OHH + E.H2/2 ...
            - T*(S.H2/2 + S.OH - S.OHH) - V -
R*T/F*log(10)*pH;
    elseif ReactionNumber == 1
        % * -> *OHH and then *OHH + *OOH -> *O + *OH +
H2O. Total is:
        % * + *OOH -> *O + *OH (no effect of V and pH!)
        dG = ZPE.OH + ZPE.O - ZPE.OOH - ZPE.V - T*(S.OH +
S.O - S.OOH - S.V);
```

```
    end
    %dG = dG * Waters;
end

% dG = dG + Waters * (E.H2O + ZPE.H2O);
end

% Constants for dG calculations

function [ZPE, S, E] = GetParams
ZPE.H2 = 0.27;
ZPE.O2 = 0.10;
ZPE.H2O = 0.57;
ZPE.V = 0;
ZPE.O = 0.04;
ZPE.OH = 0.37;
ZPE.OOH = 0.48;
ZPE.OHH =0.67;
S.H2 = 0.4/298.15;
S.O2 = 0.63/298.15;
S.H2O = 0.67/298.15;
S.V = 0;
S.O = 0;
S.OH = 0;
S.OOH = 0;
S.OHH = 0;
E.H2 = -6.774;
E.O2 = -9.871;
E.H2O = -14.225;
end


% Draw the hexagon grid and color sites

function [FigHandle] = DrawHexagons(InputGrid,
HexagonSide)

GridSize = size(InputGrid);
[X, Y] = meshgrid(1:GridSize(2),1:GridSize(1));

X = HexagonSide*X(:)*sqrt(3)/2 -
1/2*HexagonSide*Y(:)*sqrt(3)/2;
Y = sqrt(3)/2*HexagonSide*Y(:)*sqrt(3)/2;

TotX = [X X+sqrt(3)/4*HexagonSide X+sqrt(3)/4*HexagonSide
X X-sqrt(3)/4*HexagonSide X-sqrt(3)/4*HexagonSide];
```

```
TotY = [Y+HexagonSide/2 Y+1/4*HexagonSide Y-
1/4*HexagonSide Y-HexagonSide/2 Y-1/4*HexagonSide
Y+1/4*HexagonSide];

patch(TotX', TotY', InputGrid(:));

for i = 1:5
    p(i) = patch(NaN, NaN, i);
end

legend(p,'*','*OH_2','*OH','*O','*OOH');
end


% Draw a movie frame

function FigHandle = SingleHexMovieFrame(FrameMatrix,
NumbersToWrite)

f1 = figure;
DrawHexagons(FrameMatrix,10);
f1.Children(2).XAxis.Visible = 'off';
f1.Children(2).YAxis.Visible = 'off';
f1.Children(1).FontSize = 14;
f2 = figure;
C =
categorical(FrameMatrix(:),1:5,{'*','*OH_2','*OH','*O','*
OOH'});
h = histogram(C);
ylim([0 numel(FrameMatrix)]);
f2.Children.FontSize = 14;
if ~exist('NumbersToWrite','var')
    y = h.BinCounts;
    x = 0;
else
    y = NumbersToWrite;
    p = round(y/sum(y) * 100,2);
    x = 0.15;
    z = 0.27;
end
text([1:5]-0.18-x, y+90, num2str(y'),'FontSize',14)
text([1:5]-0.18-z, y+40, [repmat('(',5,1) num2str(p')
repmat('%)',5,1)],'FontSize',14)
ylabel({'Average Number of Surface Species','(Percent of
Total)'})
FigHandle = f2;
end
```

```
% Average several frames of a simulation to calculate
average coverage

function [FigHandle, AvgInterNums] =
AverageManyFrames(MovieFile, Frames)

InterNums = zeros(1,5);
for i = Frames
    CurFrame = MovieFile(:,:,i);
    for j = 1:5
        InterNums(j) = InterNums(j) + sum(CurFrame(:) ==
j);
    end
end

AvgInterNums = InterNums / numel(Frames);
AvgSums = round(cumsum(AvgInterNums));

CurFrame(1:AvgSums(1)) = 1;
CurFrame(AvgSums(1)+1:AvgSums(2)) = 2;
CurFrame(AvgSums(2)+1:AvgSums(3)) = 3;
CurFrame(AvgSums(3)+1:AvgSums(4)) = 4;
CurFrame(AvgSums(4)+1:end) = 5;

FigHandle =
SingleHexMovieFrame(CurFrame,round(AvgInterNums,2));

end



% Create a movie from simulation results

function CreateHexMovie(MovieMatrix, FileName)

% Open VideoWriter object for video writing
vid = VideoWriter(FileName);
open(vid);

h = figure('WindowState','maximized','Visible','on');

for i = 1:100:size(MovieMatrix,3)
    CurFrame = MovieMatrix(:,:,i);
    subplot(1,2,1);
    DrawHexagons(CurFrame,10);
    subplot(1,2,2);
```

```
    C =
categorical(CurFrame(:),1:5,{'*','*OH_2','*OH','*O','*OOH
'});
    histogram(C);
    ylim([0 numel(CurFrame)]);
    frame = getframe(h);
    writeVideo(vid,frame);
end

close(vid);
end
```

Reference

[1] M. C. Toroker, *J. Phys. Chem. C* **2014**, *118*, 23162–23167.
